# Co-Occurrence of Multiple Endocrine Abnormalities Induced by the DIHS/DRESS

**DOI:** 10.1155/2019/7959615

**Published:** 2019-10-03

**Authors:** Mingqun Deng, Han Wu, Miao Yu, Yi Tian, Yuxiu Li, Xinhua Xiao

**Affiliations:** Department of Endocrinology, Key Laboratory of Endocrinology, Ministry of Health, Peking Union Medical College Hospital, Peking Union Medical College and Chinese Academy of Medical Sciences, Beijing 100730, China

## Abstract

**Background:**

Drug-induced hypersensitivity syndrome/drug reaction with eosinophilia and systemic symptoms (DIHS/DRESS) is a severe adverse reaction caused by specific drugs. However, little information is available about sequelae following DIHS/DRESS resolution from an endocrinologist's perspective. This study aimed to investigate the endocrine sequelae following DIHS/DRESS, from clinical feature to etiology.

**Methods:**

We retrospectively analyzed the patients diagnosed with DIHS/DRESS in Peking Union Medical College Hospital (PUMCH) during the period of 1 January 2012 to 31 December 2017, and those who developed endocrine disorders after DIHS/DRESS were further examined. We also reviewed the literature, from 1 January 2000 to 31 December 2017, on involvement of endocrine glands in DIHS/DRESS patients.

**Results:**

Three patients developed both autoimmune thyroid disease (AITD) and type 1 diabetes (T1DM)/fulminant type 1 diabetes (FT1DM) of the 45 patients. Seven cases involving more than two endocrine glands were reported in the literature. Our results indicated that DIHS/DRESS is a potential etiological factor of autoimmune polyendocrine syndrome (APS), especially APS III.

**Conclusions:**

Patients require careful long-term follow-up after DIHS/DRESS. Involvement of endocrine glands, especially FT1DM, should always be monitored in patients with a history of DIHS/DRESS. This study indicated that DIHS/DRESS could lead to APS, especially APS III, providing novel insights into the etiological factors of APS.

## 1. Background

The drug-induced hypersensitivity syndrome/drug reaction with eosinophilia and systemic symptoms (DIHS/DRESS) is a severe adverse drug reaction. A growing number of reports have documented the occurrence of newly developed diseases after DIHS/DRESS resolution, especially autoimmune diseases such as type 1 diabetes mellitus (T1DM) [1–4] and autoimmune thyroid disease (AITD) [5–8]. Following DIHS/DRESS, fulminant type 1 diabetes mellitus (FT1DM) has been reported more often than typical T1DM. The rapid and destructive onset of FT1DM should be paid great attention. More than one autoimmune disease involving endocrine glands can co-occur in the same patient, a condition referred to as autoimmune polyendocrine syndrome (APS). However, little attention has been paid by endocrinologists to this ailment. Here, we report 3 patients with co-occurrence of FT1DM and AITD.

## 2. Methods

We reviewed the medical records of all inpatients diagnosed with DIHS/DRESS in PUMCH between 1 January 2012 and 31 December 2017. The diagnostic criteria of DRESS used in this study were proposed by European Registry of Severe Cutaneous Adverse Reactions (RegiSCAR) [9]. In January 2018, we obtained the current medical history for all patients through phone calls. Patients with suspected endocrine disorders after DIHS/DRESS were readmitted for further investigation. Follow-up data, including clinical records, physical examination, and routine laboratory examination, were obtained.

A review of the published literature on endocrine abnormalities induced by DIHS/DRESS was performed. The key terms “Drug-induced Hypersensitivity Syndrome,” “Drug-induced Hypersensitivity Syndromes,” “Drug Reaction with Eosinophilia and Systemic Symptoms,” “Drug Hypersensitivity Syndromes,” “Hypersensitivity Syndrome, Drug,” “Hypersensitivity Syndromes, Drug,” “Syndrome, Drug Hypersensitivity,” “Syndromes, Drug Hypersensitivity,” “DRESS Syndrome,” “DRESS Syndromes,” “Drug Reaction with Eosinophilia and Systemic Symptoms Syndrome,” “Autoimmune Thyroid Disease,” “AITD,” “Thyroiditis,” “Diabetes,” “Diabetes Mellitus,” and “DM” were used to search PUBMED for publications written in English on human subjects from 1 January 2000 to 31 December 2018. Studies reporting patients with more than one endocrine abnormality after DIHS/DRESS were included.

## 3. Results

Forty-five patients were diagnosed as DIHS/DRESS in our hospital during 1 January 2012 to 31 December 2017. Four individuals described that they were diagnosed with endocrine gland damage after DIHS/DRESS, and the other forty-one patients denied any symptoms of hyperglycemia, hypothyroidism, adrenal insufficiency, or hypopituitarism. One patient developed hypothyroidism, and the other three patients were diagnosed with both T1DM and Hashimoto's thyroiditis (HT). The patient with only hypothyroidism declined further investigation; the other three patients were readmitted into our department. All the three patients were demonstrated to be both T1DM (including FT1DM) and HT at readmission as presented below (Table 1). Comparing with the other forty-two patients, the average age of the three patients seemed younger (22.5 ± 3.4 vs 18.9 ± 6.0, *P*=0.074). No gender preference was revealed as the percentage of female patients was 55.6% and 70% (*P*=0.494), respectively.

### 3.1. Case 1

**Figure 1:**
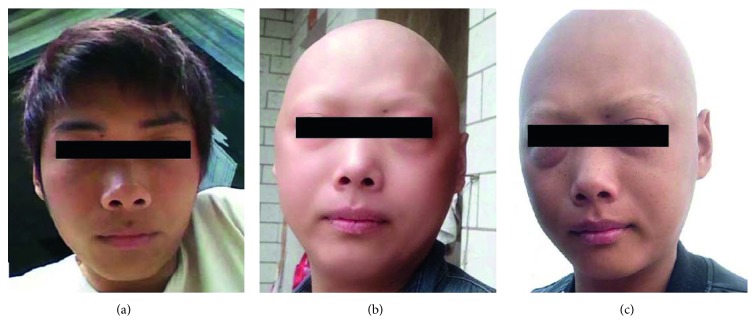
Case 1, 25-year-old male. (a) Before DIHS/DRESS; (b) July 2016, 4 months after DIHS/DRESS, the patient had no hair in the whole body; (c) September 2016, 6 months after DIHS/DRESS, eyebrow began to grow.

In March 2016, after treatment with nonsteroidal anti-inflammatory drugs (NSAIDs) and antibiotics, a 25-year-old male patient gradually experienced fever (39.8°C), with morbilliform rashes, facial edema, and generalized erythema (Figure 1). Laboratory tests showed hypereosinopenia, liver dysfunction, and acute kidney injury. Ultrasound indicated inguinal lymph node enlargement, while skin biopsy revealed exfoliative dermatitis. With irregular use of systemic glucocorticoid for about one month, which was starting with intravenous infusion of methylprednisolone 80 mg for one day and then 40 mg for two days, he gradually recovered. However, he was admitted to the Emergency Room of PUMCH because of severe ketoacidosis in May after discontinuing prednisone for 2 days. Laboratory analysis showed artery blood gas (ABG) of pH 7.064, plasma glucose of 42.4 mmol/L, glycated hemoglobin (HbA1c) of 7.8%, and fasting C peptide <0.05 ng/ml. Islet cell antibodies-IgG (ICA-IgG), glutamic acid decarboxylase antibodies (GAD), and anti-insulin-associated protein-2 Antibody (IA-2Ab) were negative, and insulin autoantibody (IAA) was positive. Rubella virus-IgG (RV-IgG), RV-IgM, and CMV-IgG were found to be positive at that time. Insulin therapy was then prescribed. In January 2017, with fatigue for 1 month, he was referred to the Endocrinology Department of PUMCH. The thyroid function test indicated primary hypothyroidism with elevated antithyroid peroxidase antibodies (A-TPO), antithyroglobulin antibodies (A-Tg), and thyrotropin receptor antibodies (TRAb). Thyroid ultrasonography showed diffuse reduced echogenicity. Therefore, a clinical diagnosis of HT was made. Fatigue was significantly improved with levothyroxine replacement therapy. No involvement of other endocrine glands was found in his readmission in January 2018.

### 3.2. Case 2

A 33-year-old female was admitted in our department in March 2018 for further clinical investigation. In October 2015, the patient had a fever of 38.8°C, rashes on the whole body, kidney injury, and hepatitis, after treatment with Chinese traditional medicine (specific ingredients were unknown). She was referred to the Dermatology Department of our hospital. Parvovirus B19 IgM was positive at that time. A diagnosis of DIHS/DRESS was made, and intravenous infusion of methylprednisolone (40 mg every day) was prescribed. In December 2015, her plasma glucose was normal. However, she suffered from nausea, vomiting, and abdominal pain in January 2016. ABG showed pH of 7.188, with plasma glucose of 33.0 mmol/L, HbA1c of 10.58%, and positive ketone bodies in urine. She began insulin therapy since then, with no more prednisone. In the further investigation in March 2018, laboratory examinations showed fasting C peptide and 2 h postprandial C peptide were all below 0.05 ng/mL. ICA-IgG and IA-2Ab were negative, while GAD was >2000 U/ml. IAA was positive, with a titer of 4.63 U/ml. The thyroid function test was normal, with overtly elevated A-TPO and A-Tg. Thyroid ultrasound showed inhomogeneous internal echoes with regions of reduced echogenicity, as well as a thyroid solid nodule with microcalcification. Fine-needle aspiration biopsy (FNAB) indicated papillary thyroid carcinoma (PTC).

### 3.3. Case 3

A 25-year-old woman was referred to the dermatology department of our hospital in June 2017. After taking oxcarbazepine for about 1 month, she suffered from a fever of 39°C, facial edema, and erythema gradually developing to the whole body. Laboratory tests indicated hypereosinopenia and atypical lymphocytes in peripheral blood. Elevated alanine transaminase (ALT) and aspartate aminotransferase (AST) indicated liver injury, while the kidney was spared. RV-IgG, CMV-IgG, and HSV-1-IgG were positive. Diagnosis of DIHS/DRESS was made by the dermatologist, and treatment with glucocorticoid was initiated as the following: intravenous infusion of methylprednisolone (40 mg every day) for five days was prescribed and then oral prednisone. While the abovementioned symptoms faded, she presented to the emergency department because of diabetic ketoacidosis (DKA) in October 2017. For further care, she was referred to our department in December 2017. HbA1c was 8.6%, with fasting C peptide and 2 h postprandial C peptide below detectable levels, ICA-IgG and IA-2Ab were negative, and GAD was positive with a low titer. IAA was also positive, with a titer of 22.22 IU/mL. After admission in our department in March 2018, thyroid function was normal with overtly elevated levels of A-TPO and A-Tg. Thyroid ultrasound showed inhomogeneous internal echoes with solid nodules.

### 3.4. Literature Review

Studies published between 1 January 2000 and 31 December 2017 were reviewed. A total of 7 cases involved more than two endocrine glands [6, 10–14] (Table 2). Of the 7 cases, 3 were FT1DM and 4 were typical T1DM. Although 1 case was diagnosed as thyroiditis with A-TPO, A-Tg, or thyroid-stimulating immunoglobulin (TSI) undetected, the remaining 6 were definite AITD.

## 4. Discussion

DIHS/DRESS is a severe adverse drug reaction. Various medications have been described to cause DIHS/DRESS; phenytoin and allopurinol are the two most common culprit drugs. DIHS/DRESS is characterized by rashes, fever, hypereosinopenia, lymph node enlargement, and visceral involvement (liver, lung, kidney, etc.) [15]. There is no diagnostic standard of DIHS/DRESS, and the diagnostic criteria from Europe and Japan are widely used in clinical practice [15, 16]. In Asia, the DIHS/DRESS represents almost one tenth of all adverse drug reaction cases, with a mortality rate of 3–10% [17–20]. Various newly developed autoimmune diseases are considered sequelae of the DIHS/DRESS, including thrombotic thrombocytopenic purpura (TTP) [21], autoimmune hemolytic anemia (AHA) [22], sclerodermoid graft-versus-host disease-like lesions [23], and systemic lupus erythematosus (SLE) [24]. FT1DM is one of the most important sequelae of DIHS/DRESS. It has been reported to occur at intervals of 39.9 days averagely after clinical resolution of the DIHS/DRESS [4]. According to the Committee of the Japan Diabetes Society, FT1DM is confirmed when all the following three findings are present [25]: (1) occurrence of diabetic ketosis or ketoacidosis soon (approximately 7 days) after the onset of hyperglycemic symptoms (elevation of urinary and/or serum ketone bodies at the first visit); (2) plasma glucose level ≥16.0 mmol/L (≥288 mg/dL) and glycated hemoglobin level <8.7% (NGSP value) at the first visit; (3) urinary C‐peptide excretion <10 *μ*g/day or fasting serum C‐peptide level <0.3 ng/mL (<0.10 nmol/L) and <0.5 ng/mL (<0.17 nmol/L) after intravenous glucagon (or after meal) load at onset. Case 1 and Case 3 could be definitely be diagnosed as FT1DM. In the current cases, FT1DM occurred between 2 and 4 months in all cases, with rapid onset of DKA. FT1DM usually occurs long time after recovery from the DIHS/DRESS and could be easily ignored without special attention from clinicians. However, its abrupt onset requires prompt intervention. Therefore, patients with DIHS/DRESS should be followed up carefully. Meanwhile, elevated thyroid-related autoantibodies were observed in all three patients. Combined with features on the ultrasound, the three patients were clinically diagnosed as HT. HT is one of the most common AITD. According to the previous studies and our cases, AITD occurs at intervals of several months to years after clinical resolution of DIHS/DRESS [26].

Regulatory T cells (Tregs), with high expression levels of CD4, CD25, and forkhead box P3 (FoxP3), are a special T-cell subpopulation, which suppresses the immune system. Actually, Tregs have been well documented to play crucial physiological roles in preventing the development of autoimmune diseases and maintaining self-tolerance [27]. In 2009, Takahashi et al. [28] firstly demonstrated that CD4, CD25, and FoxP3 Tregs expanded at the acute stage of the DIHS/DRESS. However, they become functionally deficient after DIHS/DRESS resolution. Viral reactivation plays an important role in Tregs dysfunction. The expansion of Tregs allows sequential reactivation of viruses, which in turn elicit repeated and excessive activation of antiviral T-cell responses. Tregs excessively activated to limit overactivation of antiviral T-cell responses might be eventually exhausted at the resolution stage [24]. A reduction in Tregs or an attenuation of their suppressive activity would free autoreactive T cells, which may elicit autoimmunity [29]. In conclusion, Treg dysfunction may explain why autoimmune diseases occur in patients with DIHS/DRESS, especially at the resolution stage. As for T1DM, increasing evidence indicates that Tregs play a central role in suppressing T‐cell‐mediated immune responses as well as the development of both typical T1DM and FT1DM [30]. In 2013, Haseda et al. [31] found that CD4 (+) CD45RA (−) FoxP3 highly activated Tregs are functionally impaired in both FT1DM and typical T1DM, especially with undetectable C peptide. This corroborates the notion that FT1DM is more common than the typical T1DM as a DIHS/DRESS sequela.

APS is defined as the coexistence of two endocrine autoimmune diseases [32]. Found in 70–75% of APS patients, AITD represents the most frequent autoimmune endocrinopathy. The second most common endocrine autoimmune disease in adult APS is T1DM [33]. Combination of both diseases, without Addison's disease, is termed as APS type III which represents the most prevalent subtype [34]. Brown reported a case with co-occurrence of Graves' disease and typical T1DM after DIHS/DRESS. With two autoimmune endocrinopathies, we think the patient could be diagnosed as APS III. In other words, the DIHS/DRESS syndrome could lead to APS III. By reviewing clinical records, we found that 4 of 45 cases (8.8%) develop autoimmune endocrine sequelae after the DIHS/DRESS. As we did not re-evaluate the endocrine system of all 45 patients, actual cases with endocrine glands involvement may be underestimated. Meanwhile, 3 of 4 cases involved more than one endocrine gland, indicating APS III does not seem to be rare in DIHS/DRESS. However, only a few cases with co-occurrence of T1DM and AITD have been reported by others. This might result from clinicians' ignorance to systemic evaluation of endocrine involvement. Although patients developed APS III seemed younger than those without APS III, it did not reach statistical difference. As for gender, there seemed more female patients in APS III than general DIHS/DRESS patients. However, no statistical difference reached either. It has been reported that intravenous immunoglobulin (IVIG) or pulsed prednisone can accelerate a rapid recovery of B and T cells in the acute stage of DIHS/DRESS, which might relate to the development of APS III. However, only 2 out of 7 patients in the literature were treated with IVIG, and 1 out of 7 patients were treated with pulsed prednisone. No patients who developed APS III later were treated with IVIG in the acute stage of DIHS/DRESS in our center. As discussed above, functional reduction of Tregs may contribute to autoimmune sequelae of the DIHS/DRESS syndrome. In APS III, it was found that reduced function of Tregs associated with FOXP3 genetic variants could promote the development of autoimmune endocrinopathies, especially APS III [35]. Therefore, we suppose that Tregs dysfunction bridges autoimmune sequalae of DIHS/DRESS with APS. Younger female patients with DIHS/DRESS and patients treated with pulsed prednisone or IVIG might have the greater risk for subsequently developing APS III, which needed further investigation to confirm.

There are some limitations of our study. Firstly, as most patients were from out of Beijing, we identified if patients had endocrine abnormalities through phone calls, which might result in missing cases. Secondly, some clinical information was inaccessible as a retrospective study, including serum pancreatic enzymes in the onset of FT1DM. Thirdly, we did not investigate the Tregs function in our study.

## 5. Conclusions

Patients with the DIHS/DRESS should undergo long-term monitoring for signs or symptoms suggestive of autoimmune diseases. Functional defect of Tregs after the resolution of the DIHS/DRESS may contribute to autoimmune sequelae. The present findings suggested that APS could be induced by DIHS/DRESS, especially APS III, which might be due to similar pathogenesis of Tregs. Among all autoimmune diseases, FT1DM should be extremely paid attention to, as it is not rare and may lead to severe consequences.

## Figures and Tables

**Table 1:** Three cases of co-occurrence of FT1DM and HT in PUMCH during January 2012 to December 2017.

		Case 1	Case 2	Case 3
Age		25	33	25

Gender		Male	Female	Female

DIHS/DRESS syndrome	Causative drugs	Unknown	Unknown	Oxcarbazepine
	Fever > 38.5°C	Yes	Yes	Yes
	Rashes	Yes	Yes	Yes
	Eosinophils	1.45 × 10^9^/L	5.45 × 10^9^/L	0.74 × 10^9^/L
	Other organs involvement	ALT 301 U/L, Cr 485 *μ*mol/L	ALT 206 U/L, Cr 181 *μ*mol/L	ALT 273 U/L

Systemic corticosteroid therapy (mg/day) or IVIG		Irregular use of systemic glucocorticoid for one month (started with methylprednisolone 80 mg iv qd)	Methylprednisolone 40 mg iv qd	Methylprednisolone 40 mg iv qd for 5 days, then 40 mg po qd

Diabetes mellitus	Diagnosis	FT1DM	T1DM	FT1DM
	Interval of time^#^	2 months	3 months	4 months
	Onset by DKA	Yes	Yes	Yes
	HbA1c (%)	7.8%	10.58%	8.6%
	Fasting C peptide (ng/mL)	<0.05	<0.05	<0.05
	IA-2Ab (IU/mL)	0.57	0.87	0.57
	ICA-IgG	Negative	Negative	Negative
	GAD (IU/mL)	2.33	>2000	6.00
	IAA^*∗*^ (IU/mL)	11.26	4.63	22.22

Autoimmune thyroid disease	Diagnosis	HT	HT, PTC	HT
	Interval of time	9 months	53 months	9 months
	Thyroid function test	Primary hypothyroidism	Normal	Normal
	A-Tg (IU/ml)	18.00	494.60	278.60
	A-TPO (IU/ml)	73.50	>600.00	150.40

Hypothalamic-pituitary-adrenal axis	ACTH (8AM) (pg/ml)	35.2	ND	24.4
	Cortisol (8AM) (*μ*g/dl)	18.35	ND	15.11

GH-IGF1 axis	GH (ng/ml)	0.1	ND	ND
	IGF1 (ng/ml)	252	205	236

Gonadal hormone	LH (IU/L)	7.64	11.66	4.18
	FSH (IU/L)	23.35	23.54	10.07
	T (ng/ml) or E2 (pg/ml)	4.94	45.29	59.93

Hypoparathyroidism		No	No	No

ND: no data. ^*∗*^Measured by the radioligand-binding assay (RBA); ^#^the resolution of DIHS/DRESS and the onset of DM or AITD. Normal range: IA-2Ab 0.00–1.00 IU/mL; GAD 0.00–5.00 IU/mL; IAA 0.00–0.40 IU/mL; A-Tg < 115 IU/mL; A-TPO < 34 IU/mL.

**Table 2:** Cases with more than one endocrinal disorder following DIHS/DRESS from January 2000 to December 2017.

		Case 1	Case 2	Case 3	Case 4	Case 5	Case 6	Case 7
Age		15	43	71	47	21	6	17
Gender		F	M	F	F	F	M	F
Causative drugs		Minocycline	Dapsone	Mexiletine	ND	Lamotrigine	Lamotrigine	Zonisamide
Systemic corticosteroid therapy (mg/day) or IVIG		Prednisone therapy at escalating dose from 10 mg once daily to 40 mg twice daily	A tapering dose of prednisone (unknown exact dose of prednisone)	Prednisolone, 60 mg daily	ND	Meprednisone 160 mg (40 mg every 6 hours) followed by 80 mg of prednisone; IVIG	36 mg (1.5 mg/kg/day) of prednisone; IVIG	1 g of meprednisone for 3 days followed by 45 mg of prednisone

Diabetes mellitus	Diagnosis	T1DM	FT1DM	FT1DM	FT1DM	T1DM		T1DM
	Interval of time	7 months	2 months	7 days	ND	ND	4 months	2 months
	Onset by DKA	No	No	ND	Yes	ND	Yes	ND
	HbA1c (%)	8.1%	5.9%	6.0%	6.5%	ND	10.2%	ND
	Fasting C peptide (ng/mL)	ND	ND	<0.05	0.2	ND	ND	ND
	IA-2Ab	Positive	ND	Negative	Negative	Positive	Positive	ND
	ICA-IgG	ND	Negative	Negative	Negative	ND	ND	ND
	GAD	Positive	Negative	Negative	Negative	ND	ND	ND
	IA or IAA	ND	IA is positive	ND	ND	Positive	Positive	ND

Autoimmune thyroid disease	Diagnosis	Graves' disease	Thyroiditis	HT	Painless thyroiditis	ND	HT	HT
	Interval of time	51 days	2 months	28 days	ND	8 months	4 months	2 months
	Thyroid function test	Primary hyperthyroidism	Remission of thyrotoxicosis within a few weeks	Primary hyperthyroidism	Primary hyperthyroidism	Transient hyperthyroidism and subsequent chronic hypothyroidism	ND	ND
	A-Tg	Positive	ND	Positive	ND	ND	Positive	Positive
	A-TPO	Positive	ND	Positive	ND	Positive	Positive	Positive
	TSI	Positive	Negative	ND	Negative	Positive	ND	Positive

Other involvement of endocrine glands		ND	ND	ND	ND	ND	ND	ND
Country		America	America	Japan	Japan	America	Japan	Japan
Year		2009	2017	2013	2006	2013	2018	2016
Literature								

TSI, thyroid-stimulating immunoglobulin.

## Data Availability

The datasets used and/or analyzed in the current study are available from the corresponding author on reasonable request.

## Abbreviations

DIHS/DRESS: The drug-induced hypersensitivity syndrome/drug reaction with eosinophilia and systemic symptoms
PUMCH: Peking Union Medical College Hospital
AITD: Autoimmune thyroid disease
FT1DM: Fulminant type 1 diabetes mellitus
APS: Autoimmune polyendocrine syndrome
T1DM: Type 1 diabetes mellitus
HT: Hashimoto's thyroiditis
HbA1C: Glycated hemoglobin
ABG: Artery blood gas
ICA-IgG: Islet cell antibodies-IgG
GAD: Glutamic acid decarboxylase antibodies
IA-2Ab: Anti-insulin-associated protein-2 antibody
A-TPO: Antithyroid peroxidase antibodies
A-Tg: Antithyroglobulin antibodies
TRAb: Thyrotropin receptor antibodies
FNAB: Fine-needle aspiration biopsy
PTC: Papillary thyroid carcinoma
ALT: Alanine transaminase
AST: Aspartate aminotransferase
TSI: Thyroid-stimulating immunoglobulin
TTP: Thrombotic thrombocytopenic purpura
AHA: Autoimmune hemolytic anemia
SLE: Systemic lupus erythematosus
FoxP3: Forkhead box P3
Tregs: Regulatory T cells
RV-IgG: Rubella virus-IgG
RV-IgM: Rubella virus-IgM
CMV-IgG: Cytomegalovirus-IgG
HSV-1-IgG: Herpes simplex virus type 1-IgG.

## Additional Points

*Highlights.* Patients require careful long-term follow-up after DIHS/DRESS. Functional defect of Tregs dysfunction after the resolution of the DIHS/DRESS contributes to autoimmune sequelae. The present findings suggested that APS, especially APS III, could be induced by DIHS/DRESS, which might be due to similar pathogenesis of Tregs.
